# Gradation Design and Parameter Determination of Warm-Mix-Agent-Modified Asphalt Mixture

**DOI:** 10.3390/ma15051866

**Published:** 2022-03-02

**Authors:** Junwei Xiang, Hui Wang, Yu Xiang

**Affiliations:** 1 School of Traffic and Transportation Engineering, Changsha University of Science & Technology, Changsha 410114, China; xiangjunwei2022@126.com (J.X.); wh.cs.cn@163.com (H.W.); 2 Zhongda Testing (Hunan) Co., Ltd., Changsha 410036, China; 3 School of Civil Engineering, Central South University, Changsha 410075, China

**Keywords:** EC-120, Warm mix asphalt, Gradation design, asphalt pavement, orthogonal test

## Abstract

In order to make up for inadequacies such as high energy cost in the production process and quantities of waste gas and dust release of hot-mix asphalt (HMA), warm-mix asphalt (WMA) has been developed. In this paper, the preparation process of WMA mixture is simply introduced. According to the experimental approach of asphalt binder and asphalt mixture, EC-120 is preliminarily selected as a follow-up research object after a rheological property test and a viscosity test of five kinds of warm-mix-agent-modified asphalts combined with cost analysis. A target mix proportion of SBS~AC-16 is designed, and then through the orthogonal design of the four parameters of the Marshall test of WMA mixture, such as mixing temperature, warm-mix-agent content, compaction blows, and mixing time, the best Marshall test parameters are obtained. The results show that the best parameters are 145 °C of mixing temperature, 3% of warm-mix-agent content, 75 compaction times, and 90 s of mixing time. This study can provide technical support and reference for the construction of WMA pavement in China.

## 1. Introduction

With the development of expressways, asphalt pavement has been adopted by many countries for its excellent road performance. According to the data, more than 90% of the expressways built in China are asphalt pavement. Hot-mix asphalt (HMA) is the most widely used and has good road performance [1,2]. HMA mixture refers to the mixture formed by mixing asphalt heated to about 160 °C and mineral aggregate heated to about 180 °C at high temperature. However, asphalt and mineral aggregate expose many defects at such a high temperature, which are as follows: (i) High-temperature mixing and construction environments aggravate the aging of asphalt and weaken the road performance of asphalt mixture; (ii) Short construction time always results in idle machinery and personnel and a long construction period; (iii) Harmful gas discharges from the mixture during production cause severe environmental pollution and threaten the health of construction personnel; (iv) HMA mixture reaches above 160 °C during mixing, which consumes a lot of energy [3,4,5].

Environmental pollution and energy depletion have attracted worldwide attention. In order to alleviate ecological problems, countries all over the world have set strict limits on the emissions of greenhouse gases, harmful gas, and solid dust. Among the greenhouse gases, the share of CO_2_ reaches 55%. Due to the rapid development of road construction in recent years, greenhouse-gas emissions have increased rapidly. Environmental protection has attracted more and more attention in road-traffic construction. The research data show that during the preparation of asphalt mixture, every 10 °C rise in temperature and every 1000 tons of mixture will emit an additional 900 kg of CO_2_ [6] (see Table 1).

For the negative impact of HMA mixture, it is necessary to research green, low-carbon, and environmentally friendly asphalt mixture to replace HMA in order to save energy and protect the environment without reducing the service performance of traditional HMA pavement. Based on the background above, warm-mix-asphalt (WMA) mixture gradually appears. Generally, this kind of asphalt mixture, whose construction temperature decrease ranges from 30 °C (also 20 °C) to 50 °C, is called warm-mix-asphalt mixture.

The asphalt binder used in WMA mixture is quite different from the traditional HMA mixture. For conventional polymer-asphalt modifiers with high melt viscosity, the viscosity of the modified asphalt increases, and the workability decreases. However, WMA agent can reduce the viscosity of the modified asphalt, reduce the mixing and rolling temperature of the mixture, and effectively improve the construction workability and low energy consumption [7].

There are mainly four internationally recognized production methods of warm-mix-asphalt mixture: (i) Aspha-min (sodium aluminosilicate, an artificial zeolite) can be used to make the asphalt full of foam [8]; (ii) Foaming and viscosity-reducing warm-mix technology—water mist is sprayed to the asphalt or hydrophilic material is used to generate foam in hot asphalt, which can reduce the viscosity of asphalt and the production temperature of the mixture, which can reduce CO_2_ emissions and fuel consumption by 30% [9]; (iii) Organic additive technology, after adding organic admixtures (such as wax), the viscosity of the asphalt will decrease when the temperature is higher than the melting point of the admixture, and changing the viscosity–temperature curve of asphalt and enhancing the fluidity [10]; (iv) Emulsified-asphalt warm-mixing technology—the chemical additives include emulsifiers and modifiers, the purpose of which is to improve the workability of the mixture and improve adhesion and coating ability [11]. Although warm-mix materials have broad prospects, there are still many problems: (i) The lack of corresponding design standards; (ii) The source of raw materials needs to be expanded; (iii) The cost of warm-mixing technology is relatively high; (iv) The possibility of water damage is great [12,13].

This article combines the traffic and local climate characteristics of the southern high-temperature area and focuses on five different warm-mix modified asphalts. In this article, Aspha-Min, EC-120, EC-130, Sasobit, and HPS-1 warm-mix agents were chosen and their influences on asphalt were tested through the rheological test and Marshall test. The main research contents are as follows: (i) Research and screening of the types and characteristics of warm-mix materials; (ii) Gradation design of warm-mix-agent-modified asphalt mixture and study of Marshall test.

## 2. Materials and Methods

### 2.1. Raw Materials

#### 2.1.1. Asphalt

This paper studies the uses of No. 70 A-grade matrix asphalt and SBS (I-D)-modified asphalt, which are commonly used in road engineering in China. The related technical indicators of asphalt are shown in Table 2 and Table 3.

#### 2.1.2. Warm-Mix Agent

In this paper, five warm-mix-asphalt agents are selected for experimental research: (i) the artificial synthetic zeolite Aspha-Min produced by Eurovia Services Gmb H. Bottrop in Bottrop, Germany; (ii) EC-120 warm-mix agent produced by Shenzhen Haichuan Engineering Technology Co., Ltd., Shenzhen, China; (iii) EC-130 warm-mix agent produced by Shenzhen Haichuan Engineering Technology Co., Ltd.; (iv) Sasobit, a new polyolefin asphalt universal modifier produced by Schumann-Sasol, Hamburg, Germany); (v) HPS-1, independently developed [14].

Aspha-Min is a synthetic zeolite (sodium aluminosilicate) that can fill the pitch with foam [9]. The water contained in it accounts for 21% of its volume. When the temperature reaches 85~182 °C, the water will be released. Therefore, in mixing the mixture, Aspha-Min will release water and cause the volume expansion of the binder, thereby forming foamed asphalt, improving its workability, and the asphalt can be fully used covered at a lower temperature of aggregate.

EC-120 warm-mix agent is a synthetic linear aliphatic hydrocarbon mixture with a melting point of 100 °C. When the temperature is higher than 110 °C, it can be completely dissolved in the asphalt binder and exist in the form of a grid structure without segregation. This modifier has a significant effect in reducing the high-temperature viscosity of asphalt, and the viscosity of asphalt at low temperature (60 °C) is also increased. Therefore, adding EC-120 to asphalt can not only reduce the construction mixing and compaction temperature, but also effectively improve the high-temperature stability of the asphalt mixture. The physical and chemical properties of EC-120 agent and Sasobit are shown in Table 4.

EC-130 warm-mixing agent is white powder in appearance. It is a kind of water-containing inorganic material; the water contained in it can be continuously released at about 100 °C, and it can interact with the asphalt and make it foam, thereby achieving the effect of reducing the viscosity of the asphalt, which makes the asphalt mixture evenly mixed under relatively low-temperature conditions.

Sasobit is a hard wax made by the F-T method, which is also a hydrocarbon. When the temperature is lower than 100 °C, because Sasobit forms a net-like lattice structure in the asphalt, the stability of the asphalt is improved, and the anti-rutting performance of the pavement is also increased. Tests have proved that the simple mechanically stirred Sasobit can be melted by asphalt without segregation, which can eliminate complicated processing techniques such as high-speed shear mixing. The physical and chemical indicators are shown in Table 4.

#### 2.1.3. Aggregate

Metamorphic sandstone aggregates usually have four particle sizes, 0~3 mm, 3~5 mm, 5~10 mm, and 10~18 mm. According to the Highway Engineering Aggregate Test Regulations (JTG E42-2005) of China [15], in asphalt mixtures, those with particle sizes that are larger than 2.36 mm are coarse aggregate, and those with particle sizes of less than 2.36 mm are fine aggregate. Three coarse aggregates of 3~5 mm, 5~10 mm, and 10~18 mm are used in this paper, and the fine aggregate of 0~3 mm is used. The technical requirements and test results of coarse and fine aggregate are shown in Table 5 and Table 6, respectively.

#### 2.1.4. Ground-Granulated Blast-Furnace Slag

The test results and quality requirements of the ground-granulated blast-furnace slag (GGBS) used in this paper are shown in Table 7.

### 2.2. Preparation Process of WMA

When preparing modified asphalt, compatibility is often mentioned. Compatibility belongs to the solution theory, which is based on thermodynamics. Its essence is that the dissolving substance can be added to the solvent to form a thermodynamically stable molecular dispersion system. From the concept of amorphous polymer liquid, the thermodynamic compatibility between polymers is the ability to form a homogeneous system at any ratio. Compatibility is the thermodynamic stability of a blend in terms of technology. It is dispersed after the two materials are mixed. The easier it is to disperse, the better its stability in the blend system and the better the compatibility of the two materials. Although the two materials are incompatible, their dispersion in the blend system causes “compatibility of the incompatible system”. Compatibility is not as broad as the concept of technology in a narrow sense.

Polymer-modified asphalt is incompatible thermodynamically. The preparation of polymer-modified asphalt (such as SBR and SBS) can usually be simplified into two processes: shear and mixing ripening. During shear and mixing ripening, the temperature, mixing rate, and time will affect the performance of polymer-modified asphalt. In the process of shear and mixing at high temperatures, the polymer and asphalt are broken into irregular particles and then mixed with asphalt [16]. Therefore, high-speed shear and low-speed mixing are the necessary processes in preparing excellent traditional polymer-modified asphalt. The preparation temperature of SBS is usually higher than 160 °C. In preparation, high-speed shear and low-speed stirring will consume a lot of time and energy.

In this paper, several warm-mix agents such as Aspha-min, EC-120, EC-130, Sasobit, and HPS-1 do not need to carry out the above complex preparation process. The melting points of Aspha-min, EC-120, EC-130, Sasobit, and HPS-1 are about 100 °C. As organic materials with a low melting point, when the asphalt temperature exceeds 120 °C the warm-mix agents and asphalt exist in the form of liquid, so the warm-mix agents and asphalt can be evenly integrated without segregation. Therefore, when preparing modified asphalt, these five agents need simple mechanical stirring with asphalt at 120 °C~160 °C for about 30 min [17]. The preparation of warm-mix-modified asphalt with Aspha-min, EC-120, EC-130, Sasobit, and HPS-1 is simple and easy in terms of its preparation process. It does not consume too much energy, which is also one of its advantages for engineering applications. Therefore, this study used a high-speed shear instrument to prepare warm-mix-modified asphalt. The specific preparation scheme is 6.3%, 3.5%, 6.3%, 3.0%, and 6.3% according to real construction projects and market research, respectively, for Aspha-min, EC-120, EC-130, Sasobit, and HPS-1. First, the base asphalt is melted, mixed manually for 2~3 min, and then sheared at high speed. During the test, the asphalt temperature is at about 160 °C. The high-speed shearer shears the asphalt at 2500 r/min for 30 min to obtain the required warm asphalt.

### 2.3. Experimental Methods

#### 2.3.1. Rheological Property

##### Dynamic Shear Modulus

Generally, the dynamic shear rheometer (DSR) is used to test the viscosity and elastic characteristics of the asphalt binder. The thin asphalt-binder sample is placed between the oscillating plate and the fixed plate of DSR, so the viscosity and elasticity of the asphalt sample are measured to evaluate the viscoelastic properties of asphalt.

The viscous and elastic properties of WMA are characterized by measuring the complex shear modulus G* and phase angle *δ*. Complex modulus G* shows the ability of asphalt to resist stress under repeated shearing actions; moreover, if it is big, the asphalt can have good internal stress performance. Phase angle *δ* reflects a ratio of elastic and viscous part within asphalt. If the *δ* is small, the viscous part relatively decreases and the asphalt is difficult to create a permanent deformation. [18,19,20]. 10 rad/s frequency and 12% amplitude are both kept unchanged. According to the test method named T 0619-2000 of Standard Test Methods of Bitumen and Bituminous Mixtures for Highway Engineering (JTG E20-2011) of China [21], only if the G*/sin*δ* ≥ 1.0 kPa of the original asphalt and the original warm-mix-modified asphalt can the next test be continued, and it will fail when G*/sin*δ* < 1.0 kPa. Test temperatures are set at 58 °C, 64 °C, and 70 °C, respectively.

##### Dynamic Viscosity

The 60 °C dynamic viscosity of WMA is tested according to a test method (JTG E20-2011) of China [21]. The water tank is heated to keep the temperature constant at 60 °C, and the vacuum capillary viscometer and the sample are heated in an oven at 135 °C for 30 min. The heated viscometer is placed in the container, the hot asphalt sample is injected into the capillary viscometer from tube, and then the capillary viscometer is put along with the sample back into the 135 °C oven for 10 min. Three capillaries are taken out from the oven and placed in the 60 °C water tank, then the vacuum system is connected to the viscometer and the piston is closed. The vacuum pump is turned on to bring the vacuum in the pipe to 40 kPa and the viscometer is kept in the 60 °C water tank for another 30 min; then, the valve of the pressure reducing system is opened. The interval time when the sample rises to the first marking line and the interval time when the sample reaches the marking line of 60 s are recorded. The dynamic viscosity can be calculated by the viscometer constant multiplied by the interval time that the sample reaches the marking line of 60 s.

##### Brookfield Viscosity

Combined with the actual situation of on-site construction, mixing, and transportation, the Newtonian and non-Newtonian fluid properties of WMA at a fixed stirring speed and under 105 °C, 115 °C, 125 °C, 135 °C, 145 °C, 155 °C, and 165 °C mixing temperatures are studied.

#### 2.3.2. Marshall Test

The porosity (VV), porosity (VMA), saturation (VFA), and stability of aggregate were calculated by Marshall test according to the Construction Technical Specifications for Highway Asphalt Pavement in China (JTG F40-2004) [22].

## 3. Experimental Results

### 3.1. Rheological Property of WMA

#### 3.1.1. Dynamic Shear Property of Original Asphalt

**Figure 1:**
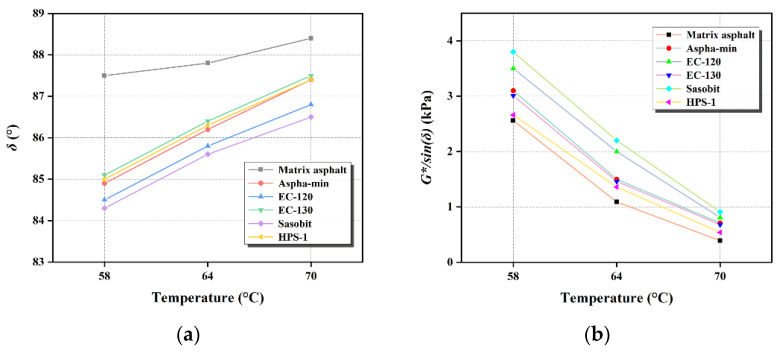
Test results of dynamic shear properties of original asphalt under different temperatures: (**a**) Phase angle; (**b**) G*/sin*δ*.

Matrix asphalt and WMA are prepared and tested, respectively. The test results are shown in Figure 1.

It can be seen from Figure 1 that the failure temperature of WMA is 70 °C. As the temperature increases, the phase angle of each material increases to different levels. At different temperatures, the phase angle of matrix asphalt is larger than other WMA. The phase angles of Sasobit and EC-120 are smaller than other warm-mix-modified asphalts. The phase angles of Aspha-min, EC-130, and HPS-1 are similar. With the increase in temperature, the phase transition of each component in the asphalt increases the viscous component, reduces the elasticity component, and then increases the phase angle of the asphalt. When the warm-mix material is composited with some components in the asphalt, the content of these components in the asphalt will also change, preventing the increase in the viscous component and the decrease in the elastic component in the asphalt, thereby limiting the rise in the phase angle of the asphalt. At high temperatures, the greater the modulus viscosity under load, the greater the inelastic deformation and the easier the asphalt will be permanently deformed. As the temperature rises, the viscous component in the viscoelasticity of the asphalt increases, and the elastic member decreases such that the phase angle of the asphalt increases.

It can be seen from Figure 1b that G*/sin*δ* (rutting factor) decreases rapidly with the temperature increase, indicating that the flow-deformation resistance of asphalt is weakened. Compared with Aspha-min, EC-120, EC-130, Sasobit, HPS-1, the rutting factor of matrix asphalt is the smallest at the same temperature, indicating that the warm-mix agent can increase the high-temperature performance of the asphalt to a certain extent and improve the ability of asphalt to resist permanent deformation. Anti-deformation capacity: Sasobit > EC-120 > Aspha-min > EC-130 > HPS-1 > matrix asphalt.

#### 3.1.2. Dynamic Shear Property of Asphalt after Short-Term Aging

**Figure 2:**
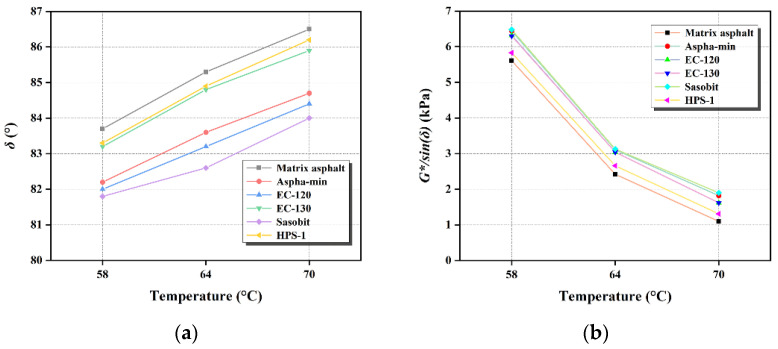
Test results of dynamic shear properties of asphalt after short-term aging under different temperatures: (**a**) Phase angle; (**b**) G*/sin*δ*.

This paper uses a rotating film oven to perform short-term aging tests on the warm-mix-agent-modified asphalt. Test results are shown in Figure 2.

It is shown in Figure 2 that the phase angle of matrix asphalt and warm-mix-agent-modified asphalt are reduced, and the addition of Aspha-min, EC-120, EC- 130, Sasobit, and HPS-1 makes the angle of WMA lower than matrix asphalt after short-term aging. At the same temperature, the phase angle of WMA is significantly lower than the matrix asphalt. Resistance to permanent deformation: Sasobit > EC-120 > Aspha-min > EC-130 > HPS-1 > matrix asphalt.

The rutting factors of Aspha-min, EC-120, EC-130, Sasobit, and HPS-1 WMA are better than the original ones after short-term aging, which indicates that the addition of warm-mix agents can delay asphalt aging and improve the high-temperature performance of asphalt.

#### 3.1.3. Dynamic Viscosity

**Figure 3:**
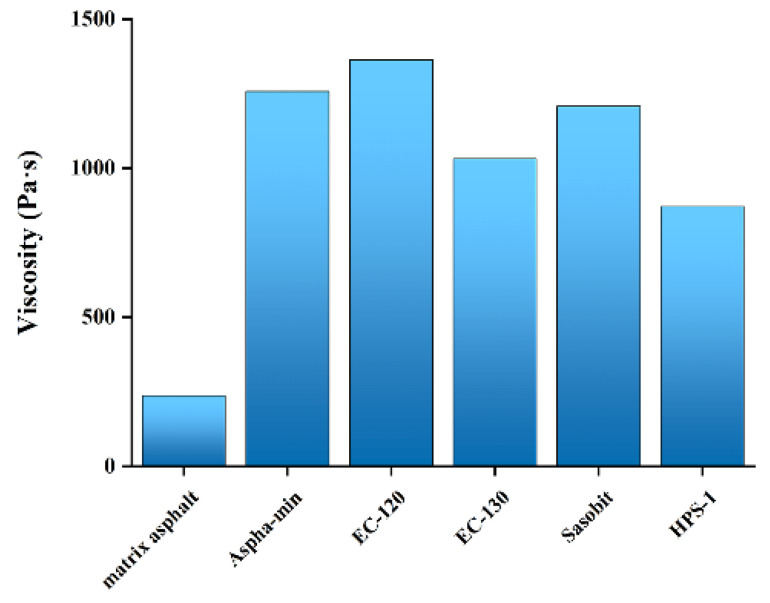
Dynamic viscosity of asphalt at 60 °C.

The matrix asphalt and WMA are prepared and tested, respectively. The dynamic viscosity results of different asphalts are shown in Figure 3.

The 60 °C viscosity index of the asphalt binder can better reflect the high-temperature anti-rutting ability of the pavement. It can be seen from Figure 3 that after adding a warm-mix agent, the viscosity is greatly improved. Asphalt with high viscosity produces less shear deformation under load and has good elastic recovery performance. EC-120 has the most significant lifting effect, and its viscosity is 5.75 times that of matrix asphalt. The viscosity of WMA mixed with Aspha-min, EC-130, Sasobit, and HPS-1 increases by 5.30, 4.35, 5.09, and 3.67 times than that of matrix asphalt, respectively. Those changes are attributed to the viscosity reducer’s high melting point. The components partially dissolved in the viscosity reducer are adsorbed by the viscosity reducer and crystallized together, which unites the saturated oil and wax components and maintains good stability.

#### 3.1.4. Brookfield viscosity

**Figure 4:**
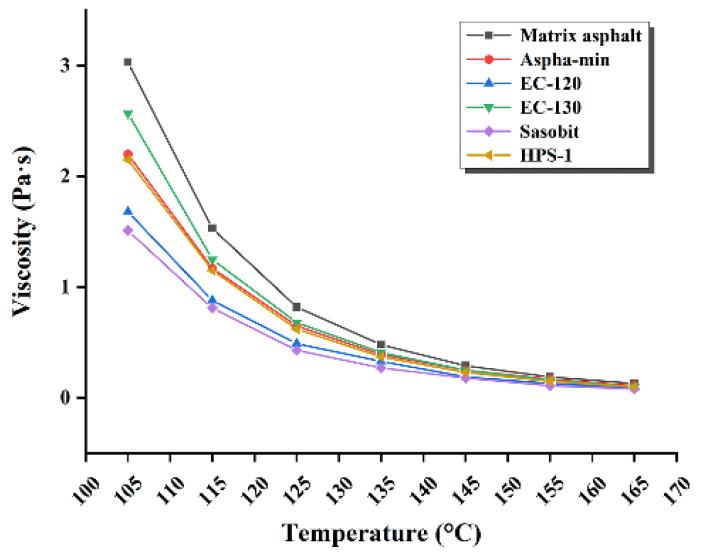
Viscosity of matrix asphalt and WMA at different temperatures.

The kinematic viscosity of matrix asphalt and WMA at different temperatures are measured by Brookfield viscometer. The test results are shown in Table 8 and Figure 4.

To ensure the ease of mixing and construction of asphalt mixtures, the standard requires that the viscosity of asphalt at 135 °C is not greater than 3 Pa∙s. It can be seen from Table 8 and Figure 4 that the viscosity of the matrix asphalt and WMA decreases with the increase in temperature, and both are lower than the matrix asphalt. The viscosities of the warm-mix-agent-modified asphalt are 43%, 54%, 39%, 47%, and 41% lower than those of matrix asphalt, respectively, which indicates that the warm-mix agent can effectively improve the workability of construction, reduce the temperature during construction, reduce energy consumption, and be beneficial to the mixing, paving, and rolling of asphalt mixtures.

### 3.2. Determination of Preparing Temperature and Modifier

Theoretically, the mixing and compaction temperature of the asphalt mixture determined by the ISO viscosity temperature is shown in Table 9.

It can be seen from Table 9 that after mixing with warm mix, all warm-mixture-modified asphalt has a lower mixing and compaction temperature than base asphalt. It can be seen from the performance analysis of the rheological performance test and viscosity study that all the warm-mix agents can improve the performance of the matrix asphalt performance. The production of 10 tons of WMA mixture can theoretically save 160,000 to 180,000 calories of heat and save 16~22 kg of standard fuel oil, which is held 16 RMB/ton. The working efficiency of the mixing plant can be increased by 20~25%, and the paving and compaction efficiency can be increased by 10~20%, thereby shortening the construction time, saving 2~5 RMB/ton of mixture and 18~21 RMB/ton of mixed material. In this study, based on the performance of various warm-mix-agent-modified asphalts, combined with the existing warm-mix market prices, EC-120 is determined as the modifier for further research after a comprehensive comparison.

### 3.3. Gradation Design and Parameter Determination of WMA Mixture

#### 3.3.1. Target Mix Proportion Design of AC-16 Asphalt Mixture

In this chapter, 3 sets of grading are designed according to AC-16 grading range and design requirements. The AC-16 asphalt-mixture mixing ratio and design gradation curve are shown in Table 10.

According to experience, for the above three gradations, 4.8% is used as the initial oil–stone ratio to conduct the Marshall test with double-sided compaction 75 times, and the physical and mechanical parameters of the specimens corresponding to each specimen are measured. The results are shown in Table 11.

**Figure 5:**
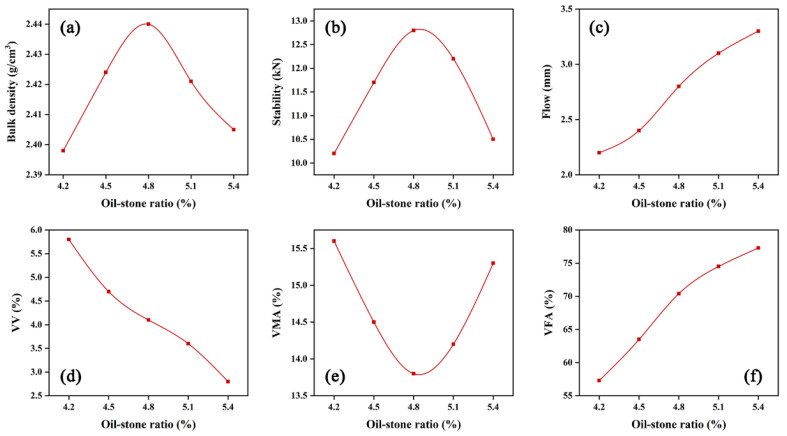
Marshall test results and volume parameters of AC-16 mixture: (**a**) bulk density; (**b**) stability; (**c**) flow; (**d**) VV; (**e**) VMA; and (**f**) VFA.

It can be seen from Table 11 that among the three gradations, the porosity and VMA of gradation 1 are too large, while the porosity and VMA of gradation 3 are too small, and the parameters of gradation 2 meet the technical requirements and the volume. The parameters of gradation 2 are more reasonable, so gradation 2 is recommended as the design gradation of AC-16 modified asphalt mixture. It is proposed that 4.8% is the initial oil–stone ratio, and five oil–stone ratios are determined according to the median value and the median value ± 0.3%, namely 4.2%, 4.5%, 4.8%, 5.1%, and 5.4%, corresponding to 4 specimens for each oil–stone ratio. Volume parameters calculate the VMA, VV, VFA, and other volume indicators of samples tested. The results are shown in Figure 5.

There is no peak density within the selected asphalt aggregate ratio, so the target porosity (4.0%) corresponds to optimum asphalt content (OAC) OAC_1_ = 4.8%, through the oil–stone ratio and Marshall index (Porosity 3~5%, VFA 65~75%), OAC_min_ = 4.6%, OAC_max_ = 5.0%, OAC_2_ = 4.8%, then the result of OAC = 4.8%. The target porosity for VMA of 4.0% is ≥ 13.5%. In the test, when the oil-to-stone ratio is 4.8%, the corresponding VMA is 13.9%, which meets the requirements of the standard, and the optimal oil–stone ratio for the target mix design is finally determined to be 4.8%.

#### 3.3.2. Marshall Orthogonal Experimental Design

Because there is no mature design standard for WMA mixture in China, many factors could affect the investigated indexes in the Marshall test. Therefore, this study uses an orthogonal experimental design to analyze the Marshall indexes and volume parameters of WMA mixture and study the influence of various factors. The orthogonal experiment can significantly reduce the number of investigations by arranging experiments through an orthogonal table. In this study, the orthogonal table L16 (4^5^) is selected.

##### Determination of Factors and Levels of Orthogonal Test

(i) Influencing factors

In the Marshall test of asphalt mixture, the determination of mixing temperature is essential. Therefore, this paper selects the mixing temperature (a) as the first factor of the orthogonal experiment. After determining the warm-mix agent, different content may show another performance, so the warm-mix agent content (b) is selected as the second factor. The Code for Design of Highway Asphalt Pavement in China stipulates that the Marshall compaction test of the high-grade highway standard has 75 compaction tests on both sides. However, the current standards in China use the Marshall design method to control the gradation of mineral materials and the amount of asphalt to design the mixture. For asphalt roads with different structural layers, working temperature, traffic volume, climate factors, and regional differences, the requirements for temperature stability of high asphalt are different. Because of the Superpave idea, the influence of compaction work on the volume index and stability of asphalt mixture is explored by analyzing the voids and stability of asphalt mixture by increasing or reducing compaction blows. Therefore, the number of double-sided compactions (c) is selected as the third influencing factor; the mixing time (d) can reflect the combined speed of warm-mix agent with asphalt and aggregate.

(ii) Selection of influencing-factor level

Four levels are set for each factor, in which the mixing temperatures (a) of EC-120 warm-mix agent are 125 °C, 135 °C, 145 °C, and 155 °C, respectively; the additions (b) of warm-mix agent are 2.5%, 3.0%, 3.5%, and 4.0% of asphalt dosage, respectively; the compaction blows (c) are 45, 55, 65, and 75, respectively; and the mixing times (d) are 60 s, 70 s, 80 s, and 90 s, respectively.

##### Determination of Test Scheme

Table 12 is the orthogonal grading design test table.

##### Orthogonal Test Results

Marshall test results and volume parameters are shown in Table 13.

## 4. Discussion

Range analysis is a simple, easy-to-understand, and intuitive method in orthogonal tests. In the orthogonal test, the range is used to judge the primary and secondary factors and the optimal level and select the optimal combination. The method used for range analysis is the R method. For the range of factors R=max(K1,K2,K3,K4…Kn)−min(K1,K2,K3,K4…Kn), the influence degree of each factor on its indicators is evaluated according to the range. Each factor has *n* levels. The *Kn* value is the average value of the sum of the values corresponding to the *n* level of each factor in the orthogonal test. The large range of the *K* value indicates that there is a large difference caused by this factor within the variation range of this level and has a great impact on the test results, so this factor becomes the main influencing factor. By analyzing the influence degree of each element, the optimal factor combination is selected.

### 4.1. Porosity Range

In the design of asphalt pavement, the porosity directly affects the rut depth, water permeability, bending stiffness, fatigue life, and strength of the asphalt pavement. Excessive porosity will accelerate aging, weaken water permeability, and show peeling of asphalt. Therefore, porosity is a crucial index in the design of asphalt pavement. For the Marshall void ratio of the orthogonal test, the range analysis is performed, as shown in Table 14.

The conclusions obtained from Table 14 are as follows:

The range R of factor (a) mixing temperature is the largest, which indicates that the mixing temperature has the most significant influence on the porosity of the mixture. Sorted according to the degree of influence of each factor on the porosity, mixing temperature (a) > compaction blows (c) > mixing time (d) > warm-mix-agent content (b).

The extreme difference in R between the mixing temperature (a) and the number of compactions (c) is greater than the blending amount of the warm-mix agent (b) and the mixing time (d). This is because the mixing temperature and the compaction work are both correct. Marshall test porosity strongly influences external factors, and the level K value of each element is different. Under the condition that the porosity that meets the standard requirements is 3~5%, for the mixing temperature (a), the K1 value is the largest, indicating that the porosity is the largest at 125 °C. When the temperature is 125 °C, it can only meet the requirements of the standard when the other three factors are the largest: the content of the warm-mix agent (b) is 4.0%, the number of compactions (c) is 75, and the mixing time (d) is 90 s. It meets the requirements of the standard. Using 4.0%—the median of the required porosity of 3~5%—as the control index, it can be concluded that K3 is the closest. This also verifies the mixing and compaction temperature based on the viscosity. Therefore, the porosity-range analysis recommends 145 °C as the mixing temperature.

For the blending amount of warm-mix agent (b), the void ratio decreases with the increase in the blending amount. The warm-mix agent has the most negligible influence on porosity among the four factors. When the warm-mix agent is 2.5%, the K value is the smallest, and the porosity is closer to the median value of 4.0%. Therefore, the recommended addition of warm-mix agent is 2.5%. Although this is somewhat different from the 3.5% blending agent recommended by the manufacturer, the 16 tests are only a tiny part of the overall possible conditions so it may be expanded. The specific dosage needs to be further verified in subsequent experiments.

The R value of the compaction work is second only to the mixing temperature among the four factors, and the K value is the smallest at 75 times. This is because the increase in the number of compactions can make the Marshall specimens more compact, which is comparable to hot mixing. Asphalt mixture meets the standard requirements, so the recommended compaction time (c) is 75. The mixing time (d) has a small R value among the four factors. The K value is the smallest when the mixing time is 90 s. The longer the mixing time, the more the warm-mix agent can be more evenly dispersed in the asphalt mixture, so the recommended mixing time is 90 s.

### 4.2. Marshall Range

The Marshall stability represents the high-temperature performance of the asphalt mixture. Therefore, the extreme analysis of the Marshall stability of the orthogonal test is carried out, as shown in Table 15.

The conclusions obtained from Table 15 are as follows.

In the range R of factor (a), the mixing temperature is the largest, indicating that the mixing temperature has the greatest influence on the stability of the mixture. Sorted according to the influence of each factor on the stability, mixing temperature (a) > mixing time (d) > compaction blows (c) > warm-mix-agent content (b).

The extreme difference in R of the mixing temperature (a) is greater than the content of the warm-mix agent (b), the number of compactions (c), and the mixing time (d), because the mixing temperature has a more significant influence on the Marshall stability. Under the condition that the stability requirement exceeds 8 kN, the mixing temperature (a) has the smallest value of K1. At 125 °C, except for the fourth group of tests, the other three groups—regardless of the factors (b), (c), and (d)’s change and stability—do not meet the requirements. Regarding the standard need for stability more remarkable than 8 kN as the control index, K3 is the largest among the factors of mixing temperature (a), so the mixing temperature is determined to be 145 °C.

The value of K is the largest when the blending amount of the warm-mix agent (b) is 2.5%, so the recommended blending amount of the warming agent is 2.5%, which is also less than the 3.5% warm-mix agent recommended by the manufacturer; however, the 16 trials of the orthogonal experiment only represent part of the working conditions, so the dosage may be expanded in other situations.

The optimal compaction work (c) level K value is K4. That is, the number of compactions is 75. The mixing time (d) and the maximum K value when the mixing time is 90 s can maximize the stability of the asphalt mixture.

### 4.3. Analysis of the VMA

The VMA of asphalt mixture has a great influence on its durability, strength, and high-temperature stability. The value of VMA can neither be too large nor too small in an interval. Therefore, when designing the asphalt mixture, a lower limit of the VMA is generally set. The void ratio of the asphalt mixture in this paper is 3~5%. Therefore, according to the Technical Standard for Highway Asphalt Pavement Construction (JTG F40-2004) of China, the VMA is not less than 14%. The range analysis is shown in Table 16.

The conclusions obtained from Table 16 are as follows.

The VMA of mineral aggregate meets the requirements. The range R of mixing temperature is the largest, indicating that the mixing temperature has the greatest influence on the VMA. According to the influence of various factors on VMA, the mixing temperature (a) > compaction blows (c) > addition of warm mix agent (b) > mixing time (d).

Mixing temperature (a) has the most significant impact on the VMA. The VMA decreases with the increase in mixing temperature. When the VMA is ≥ 13.5%, although there is no upper limit for the VMA required by the standard, too large a VMA will cause too sizeable a residual air void of asphalt mixture. Therefore, it is generally considered that it is reasonable to increase the lower limit value of the VMA standard by 0.5%; therefore, the recommended mixing temperature is 145 °C.

When the content of the warm-mix agent (b) is 3.0%, the value of K is the largest, so it is recommended that the content of the warm-mix agent be 3.0%.

When the number of compaction blows is 55, the VMA is the largest. When the VMA is 3~5%, only 75 compaction blows meet the standard requirements. Therefore, it is recommended that the number of compaction blows is 75. When the mixing time is 90 s, the value of K is the largest, making the Kn of the asphalt mixture the largest and thus the VMA meets the standard requirements. Therefore, the recommended mixing time is 90 s.

In summary, based on the range analysis, it is concluded that the optimal levels of each factor are for the mixing temperature to be 145 °C, the addition of warm-mix agent to be 3.0%, the number of compaction blows to be 75, and the mixing time to be 90 s.

## 5. Conclusions

In this paper, combined with domestic and foreign research results, the preparation process and comparison of existing warm-mixing agents are carried out, and EC-120 is selected from many warm-mixing agents in combination with three indexes: rheological properties, viscosity, and other performance parameters. Orthogonal experiments are designed for the Marshall test of SBS~WAC-16(E) asphalt mixture, and the best mixing temperature, the best warming-agent content, and the best mixing time were obtained. This article mainly evaluates several warm-mixing agents applied in real engineering through the rheological test and Marshall test, and the main purpose is to serve the actual engineering. The conclusions are as follows:

1. The test results of three indicators show that the five warm-mixing agents—Aspha-min, EC-120, EC-130, Sasobit, and HPS-1—can improve the essential performance of asphalt, reduce the penetration of asphalt, increase the softening point, and improve the high-temperature performance of the asphalt. They can also reduce the low-temperature ductility of the asphalt to a certain extent.
2. According to DSR test results, all warming-mixing agents chosen in this paper have the positive effects on asphalt. The complex modulus G* and rutting factor G*/sin*δ* of the asphalt are improved to varying degrees. The resistance to permanent deformation is enhanced, and the high-temperature performance and the ability to delay the aging of asphalt have improved.
3. The viscosity test shows that the warm-mix agent can effectively increase the viscosity of the asphalt at 60 °C and improve the resistance to shear deformation of the asphalt. At 135 °C, the order of viscosity-reduction degrees is Sasobit > EC-120 > HPS-1 > Aspha-min > EC-130, indicating that the warm-mix agent is beneficial to reduce the mixing temperature and paving temperature of the asphalt mixture. Finally, EC-120 was used for further research.
4. The best oil–stone ratio is 4.8%. The orthogonal test design method is used to determine and verify the optimal mixing temperature, the optimal amount of warm mixing agent, the optimal mixing time, and the optimal compaction blows of WMA. Marshall test shows that the results are 145 °C, 3%, 75 times, and 90 s, respectively.

## Figures and Tables

**Table 1:** Relationship between CO_2_ emission and temperature during asphalt-mixture production.

Mixing Temperature (°C)	130	140	150	160	170	180
**CO_2_ emission (kg/T)**	15.9	16.7	17.6	18.5	19.4	20.3

**Table 2:** Main indexes of No. 70 A-grade matrix asphalt.

Item		Unit	Indicator	Result	Method
Penetration (25 °C, 100 g, 5 s)		0.1 mm	60~80	70.5	T0604
Penetration Index		-	−1.5~+1.0	−0.77	T0604
Softening point (ring-ball)		°C	≥46	46	T0606
Ductility (10 °C)		cm	≥20	32	T0605
Ductility (15 °C)		cm	>100	>100	T0605
Density (25 °C)		g/cm^3^	tested	1.02	T0603
Dynamic viscosity (60 °C)		Pa∙s	>180	213	T0625
Solubility (trichloroethylene)		%	≥99.5	99.8	T0607
Flash point		°C	≥260	288	T0611
Wax content (distillation)		%	≤2.2	1.2	T0615
TFOT	Mass loss	%	±0.8	0.12	T0609
or	Penetration ratio	%	≥61	71	T0604
RTFOT	Ductility (10 °C)	cm	≥6	18	T0605

**Table 3:** Main indexes of SBS (I-D)-modified asphalt.

Item		Unit	Indicator	Result	Method
Penetration (25 °C, 100 g, 5 s)		0.1 mm	40~60	50	T0604
Penetration Index		-	≥0	1.21	T0604
Softening point (ring-ball)		°C	≥60	80.5	T0606
Ductility (15 °C)		cm	≥20	30	T0605
Dynamic viscosity (135 °C)		Pa∙s	≤3	2.3	T0625
Dynamic viscosity (60 °C)		Pa∙s	≥6000	7000	T0620
Flash point		°C	≥230	309	T0611
Solubility (trichloroethylene)		%	≥99	99.64	T0661
Elastic recovery (25 °C)		%	≥75	92	T0662
TFOT	Mass loss	%	−1.0~+1.0	0.05	T0609
or	Penetration ratio	%	≥65	76	T0604
RTFOT	Ductility (5 °C)	cm	≥15	21	T0605

**Table 4:** Basic properties of EC-120 agent and Sasobit.

Item	Freezing Point/°C	Flash Point/°C	Melting Point/°C	135 °C Viscosity/cp	25 °C Needle Penetration/0.1 mm	25 °C Density/g/cm
EC-120	97	290	100	100	<1	0.94
Sasobit	100	290	12	<1	0.94	0.94

**Table 5:** Coarse-aggregate test results.

Item		Unit	Result	Requirement
Crushing value		%	18.9	≤26
Los Angeles abrasion loss		%	15.9	≤28
Adhesion between coarse aggregate and warm-mix-modified asphalt		Level	5	5
Apparent relative density	10 mm–18 mm	-	2.771	≥2.6
	5 mm–10 mm		2.776	
	3 mm–5 mm		2.775	
Bulk-volume relative density	10 mm–18 mm	-	2.741	-
	5 mm–10 mm		2.736	
	3 mm–5 mm		2.731	
Water absorption	10 mm–18 mm	%	0.41	≤2.0
	5 mm–10 mm		0.51	
	3 mm–5 mm		0.60	
Needle-flake-particle content	10 mm–18 mm	%	5.5	≤10
	5 mm–10 mm		13.7	≤15
Ruggedness		%	0.82	≤12

**Table 6:** Technical requirements and test results of fine aggregate.

Items	Apparent Relative Density	Bulk-Volume Relative Density	Water Absorption/%	Sand Equivalent/%	Angularity/s	Mud Content/%	Ruggedness/%
Test results	2.779	2.730	0.65	63	36	2.7	2.14
Requirement	≥2.5	-	≤2	≥60	≥30	≤3	≤12

**Table 7:** Test results of mineral powder.

Index	Apparent Density/t/m^3^	Moisture Content/%	Hydrophilic Coefficient/%	Appearance	Particle Size		
					<0.6 mm	<0.15 mm	<0.075 mm
Test results	2.737	0.28	0.71	Qualified	100	96.7	83.7
Requirements	≥2.5	≤1	<1	No agglomeration	100	90–100	75–100

**Table 8:** Viscosity test results of different asphalts (Pa∙s).

Asphalt	Temperature (°C)						
	105	115	125	135	145	155	165
Matrix	3.03	1.53	0.82	0.48	0.29	0.19	0.13
Aspha-min	2.2	1.17	0.65	0.39	0.25	0.17	0.11
EC-120	1.68	0.88	0.49	0.33	0.19	0.13	0.09
EC-130	2.57	1.25	0.68	0.41	0.25	0.16	0.1
Sasobit	1.51	0.81	0.43	0.27	0.18	0.11	0.08
HPS-1	2.15	1.15	0.62	0.37	0.23	0.15	0.1

**Table 9:** Mixing and compaction temperature of asphalt.

Asphalt	Temperature (°C)					
	Lower Limit of Mixing Temperature	Upper Limit of Mixing Temperature	Mixing Temperature	Lower limit of Compacting Temperature	Upper Limit of Compacting Temperature	Compacting Temperature
Matrix	157	163	160 ± 3	142	148	145 ± 3
Aspha-min	152	158	155 ± 3	132	138	135 ± 3
EC-120	137	143	140 ± 3	122	128	125 ± 3
EC-130	152	158	155 ± 3	132	138	135 ± 3
Sasobit	142	148	145 ± 3	132	138	135 ± 3
HPS-1	152	158	155 ± 3	132	138	135 ± 3

**Table 10:** Grading range and design grading curve.

Sieve (mm)	Gradation			The Upper Limit of the Standard	The Lower Limit of the Standard	Medium of Standard
	1	2	3			
19.00	100.00	100.00	100.00	100.00	100.00	100.00
16.00	98.34	98.49	98.63	95.00	100.00	97.50
13.20	79.94	81.69	83.43	70.00	90.00	80.00
9.50	54.90	58.75	62.61	50.00	70.00	60.00
4.75	28.47	32.46	36.45	26.00	44.00	35.00
2.36	22.57	25.85	29.27	18.00	35.00	26.50
1.18	17.99	20.15	22.68	15.00	29.00	22.00
0.60	14.39	15.65	17.46	12.00	23.00	17.50
0.30	11.29	11.80	12.99	8.00	18.00	13.00
0.15	8.52	8.44	9.13	6.00	13.00	9.50
0.075	5.97	5.55	5.84	4.00	8.00	6.00

**Table 11:** Results of Marshall test of 3 gradations.

Gradation	Number	Bulk Density	VV(%)	VMA (%)	VFA (%)	Stability (kN)
1	1	2.433	4.40	14.10	68.80	15.62
	2	2.430	4.50	14.20	68.30	16.24
	3	2.423	4.80	14.40	66.70	17.02
	Average	2.429	4.57	14.23	67.93	16.29
2	1	2.444	3.90	13.70	71.50	16.24
	2	2.438	4.20	13.90	69.80	15.62
	3	2.438	4.20	13.90	69.80	16.22
	Average	2.440	4.10	13.83	70.37	16.03
3	1	2.445	3.90	13.70	71.50	16.44
	2	2.449	3.70	13.50	72.60	18.32
	3	2.457	3.40	13.20	74.20	17.23
	Average	2.450	3.67	13.47	72.77	17.33

**Table 12:** Marshall orthogonal test scheme of EC-120-modified WMA mixture.

Number	Parameters			
	a	b	c	d
1	125	2.5	45	60
2	125	3.0	55	70
3	125	3.5	65	80
4	125	4.0	75	90
5	135	2.5	55	90
6	135	3.0	45	80
7	135	3.5	75	70
8	135	4.0	65	60
9	145	2.5	65	70
10	145	3.0	75	60
11	145	3.5	45	90
12	145	4.0	55	80
13	155	2.5	75	80
14	155	3.0	65	90
15	155	3.5	55	60
16	155	4.0	45	70

**Table 13:** Orthogonal test results.

Number	Parameters		
	VV (%)	Stability (Kn)	VMA (%)
1	6.67	11.35	16.66
2	6.57	10.93	16.55
3	6.14	11.69	16.16
4	4.91	12.91	15.06
5	5.15	14.68	15.64
6	6.09	13.06	16.14
7	4.56	13.21	14.75
8	4.58	14.16	14.73
9	4.36	14.19	14.57
10	4.32	14.48	14.54
11	4.95	13.70	15.10
12	4.01	14.31	14.26
13	3.64	14.81	13.92
14	4.83	13.53	14.99
15	4.36	13.30	14.57
16	5.42	13.01	15.48

**Table 14:** Porosity-range analysis.

Porosity Factor	a	b	c	d
K1	6.07	4.95	5.78	4.98
K2	5.09	5.20	5.02	5.22
K3	4.41	5.00	5.00	4.97
K4	4.56	4.97	4.35	4.96
R	1.66	0.25	1.43	0.26

**Table 15:** Stability-range analysis.

Stability Factor	a	b	c	d
K1	11.72	13.76	12.78	13.32
K2	13.78	12.99	13.31	12.83
K3	14.17	12.98	13.39	11.97
K4	13.65	13.59	13.85	13.70
R	2.45	0.78	1.07	1.73

**Table 16:** VMA range analysis.

VMA Factor	a	b	c	d
K1	16.11	15.20	15.72	15.13
K2	15.32	15.56	15.26	15.34
K3	14.64	15.15	15.11	15.12
K4	14.62	14.88	14.57	15.20
R	1.49	0.67	1.15	0.22

## Data Availability

Data are available on request due to restrictions, e.g., privacy or ethical.
